# Reversal of Fatty Liver With Regression of Acute Necrotizing Pancreatitis: A Rare Case

**DOI:** 10.7759/cureus.65729

**Published:** 2024-07-30

**Authors:** Sanjay M Khaladkar, Sayali Paidlewar, Purnachandra Lamghare, Ankita Pandey

**Affiliations:** 1 Radiology, Dr. D. Y. Patil Medical College, Hospital and Research Centre, Dr. D. Y. Patil Vidyapeeth (Deemed to be University), Pune, IND

**Keywords:** metabolic diseases, reversal, fatty liver disease, inflammation, pancreatitis

## Abstract

Acute pancreatitis is a severe inflammatory condition that can lead to systemic repercussions, one of which is the development of hepatic steatosis (fatty liver). The accumulation of fat in liver cells can complicate the course of pancreatitis, exacerbating inflammation and causing additional metabolic disturbances. The presence of fatty liver in the context of acute pancreatitis can thus worsen the overall clinical picture, making management more challenging and potentially leading to further complications. Here, we discuss a rare case of a 34-year-old female who demonstrated the reversal of fatty liver following the improvement of acute pancreatitis. This case highlights the dynamic relationship between acute pancreatitis and hepatic steatosis, illustrating that effective management of pancreatitis can lead to significant improvements in associated conditions such as fatty liver.

## Introduction

Fatty liver disease (FLD) and acute necrotizing pancreatitis (ANP) are two distinct but serious conditions that require careful management to mitigate their impacts on health [1]. FLD, characterized by excessive fat accumulation in liver cells, can lead to inflammation, scarring (cirrhosis), and liver dysfunction if left untreated [2,3]. It often results from lifestyle factors such as excessive alcohol consumption, obesity, insulin resistance (often associated with diabetes), or a combination of these factors. Treatment typically involves lifestyle changes such as adopting a balanced diet, exercising regularly, managing weight, and abstaining from alcohol [4]. Monitoring liver function through blood tests and imaging helps assess progress and adjust treatment as needed. In more severe cases, medications to manage underlying conditions such as insulin resistance or vitamin E supplements may be prescribed.

ANP is a severe and potentially life-threatening condition characterized by inflammation and necrosis of the pancreatic tissue [5]. This condition often results from various etiologies, including gallstones, chronic alcohol consumption, or idiopathic causes. The necrosis of pancreatic tissue can lead to the release of digestive enzymes and inflammatory mediators, causing extensive damage not only to the pancreas but also to surrounding tissues and organs [6]. The systemic inflammatory response triggered by ANP can result in multi-organ failure, making early diagnosis and management critical.

The clinical presentation of ANP is typically marked by sudden onset of severe abdominal pain, nausea, vomiting, and abdominal distension [7,8]. In severe cases, patients may develop complications such as peripancreatic fluid collections, pancreatic pseudocysts, ascites, pleural effusions, and vascular complications such as splenic vein thrombosis [9]. These complications can significantly impact patient outcomes, increasing morbidity and the need for intensive care. Imaging studies, particularly contrast-enhanced computed tomography (CECT) scans, play a crucial role in diagnosing ANP and assessing the extent of pancreatic necrosis and associated complications, guiding the management plan.

The pathophysiology of fatty liver in the context of acute pancreatitis involves several mechanisms. Inflammatory cytokines released during pancreatitis can impair lipid metabolism, leading to fat accumulation in the liver [10]. Oxidative stress, a hallmark of acute pancreatitis, further exacerbates liver damage by disrupting normal lipid processing [11]. Additionally, systemic inflammation associated with pancreatitis can alter glucose and lipid metabolism, contributing to hepatic steatosis. Understanding these mechanisms is crucial for developing targeted treatments that address both pancreatic and liver inflammation.

## Case presentation

**Figure 1:**
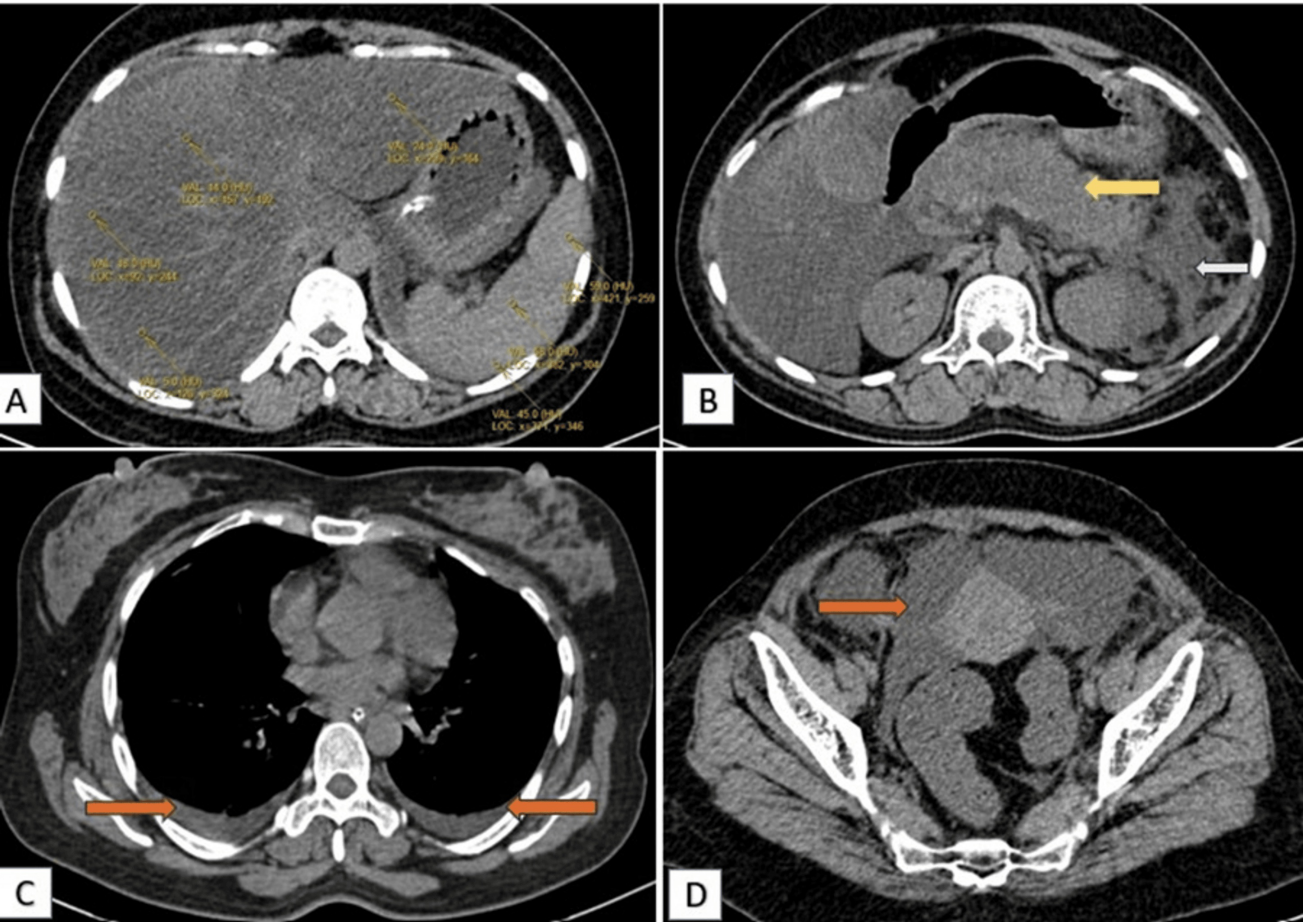
Axial plain CT images depicting changes in fatty liver with acute pancreatitis (A) Axial plain CT images depict relative hypoattenuation of the liver, liver attenuation less than the spleen (changes of fatty liver). (B) The pancreas appears bulky (yellow arrow) with peripancreatic fat stranding and reactive thickening of the left lateral conal fascia (white arrow). (C) Bilateral pleural effusion (orange arrow). (D) Free fluid seen in the pelvis region (orange arrow). CT: computed tomography

**Figure 2:**
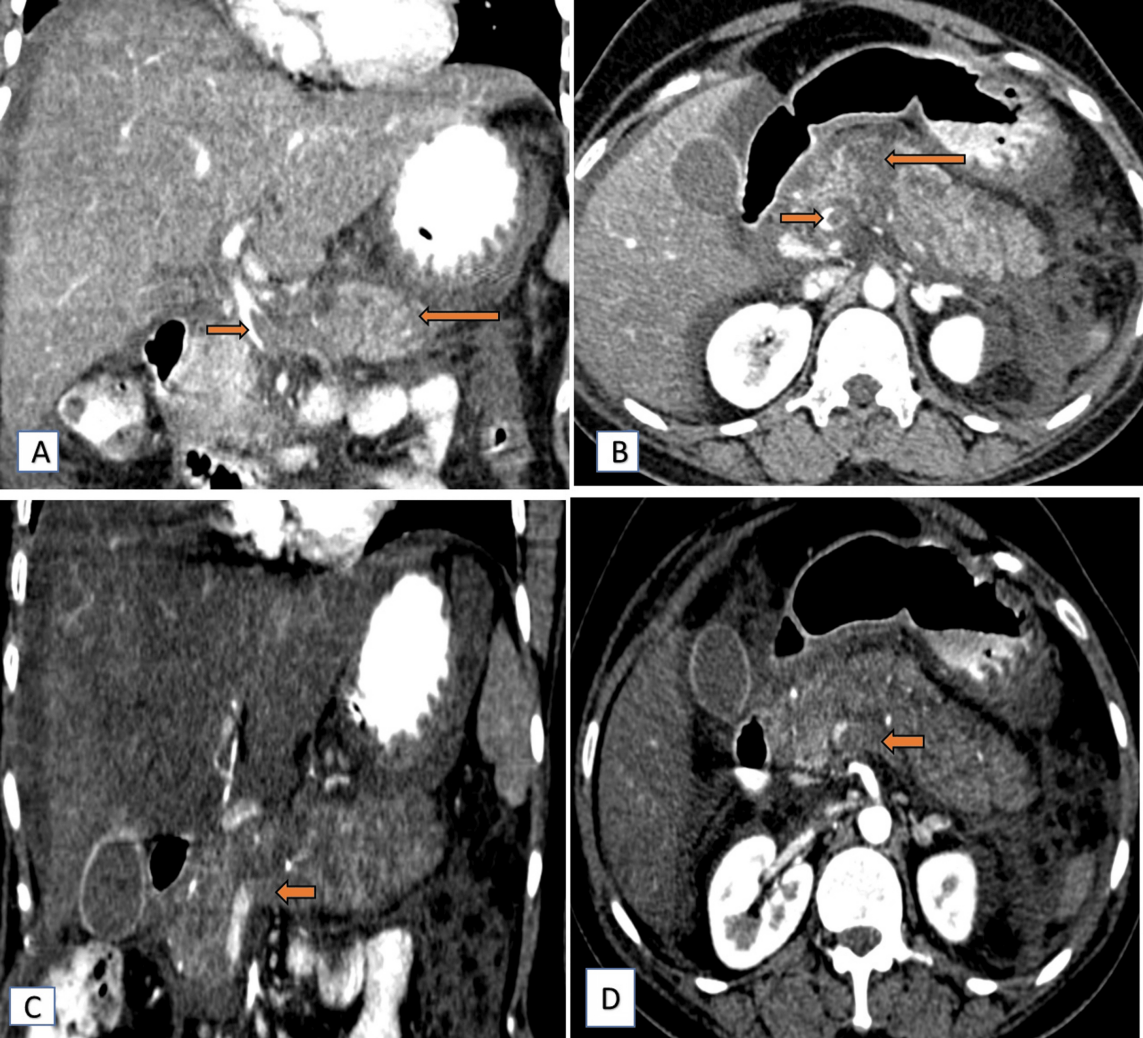
Contrast CT images (coronal and axial) showing thrombosis in the portal vein and splenic vein Contrast CT images (portal phase) reveal a hypodense filling defect in the portal vein at the region of the portal vein confluence in coronal (short arrow) (A) and axial (short arrow) (B) sections. It also shows heterogeneous pancreatic parenchymal attenuation with a few small, hypodense, non-enhancing areas in coronal (long arrow) (A) and axial (long arrow) (B) sections, suggestive of necrotic areas. Contrast CT images (arterial phase) reveal a complete non-opacification of the splenic vein in coronal (short arrow) (C) and axial (short arrow) (D) sections. CT: computed tomography

A 34-year-old female presented to the emergency department with acute abdominal pain and distension. A computed tomography (CT) scan of the abdomen and pelvis revealed significant hypoattenuation of the liver (in comparison to the spleen) (Figure 1A), signs indicative of acute necrotizing pancreatitis (ANP) (Figure 1B) with peripancreatic fat stranding and surrounding inflammatory changes in the form of reactive thickening of the left lateroconal fascia (Figure 1B). On post-contrast scans, the pancreas appeared heterogeneous with a few small, non-enhancing, hypodense areas (long arrow, Figure 2A, 2B), contributing to a modified CT severity index of 8/10 [5]. Thus, a severe form of pancreatitis is characterized by pancreatic tissue necrosis and a systemic inflammatory response. Bilateral pleural effusion and ascites (Figure 1C, 1D) were also noted. Furthermore, post-contrast images indicated hypodense filling defects in the portal vein and splenic vein, suggestive of thrombosis (Figure 2).

The initial management of the patient included conservative measures aimed at stabilizing her condition. She was administered intravenous fluids (dextrose normal saline at 80 mL/hour) to maintain hydration and electrolyte balance. Pain management was provided with injection tramadol on an as-needed basis, and antiemetic therapy was given using injection Emeset (ondansetron) 4 mg three times daily. Additionally, proton pump inhibitor therapy was initiated with injection pan (pantoprazole) 40 mg once daily to reduce gastric acid secretion and protect the gastrointestinal mucosa.

Given the severity of her condition, broad-spectrum antibiotic therapy was started with injection Monocef (ceftriaxone) 1 g twice daily and injection meropenem 1 g three times daily to combat potential infections and prevent septic complications. The presence of splenic vein thrombosis warranted the use of anticoagulant therapy, and she was prescribed tablet rivaroxaban 20 mg once daily to prevent further thrombotic events. The patient's management also included close monitoring of vital signs, laboratory parameters, and clinical status to assess her response to treatment.

**Figure 3:**
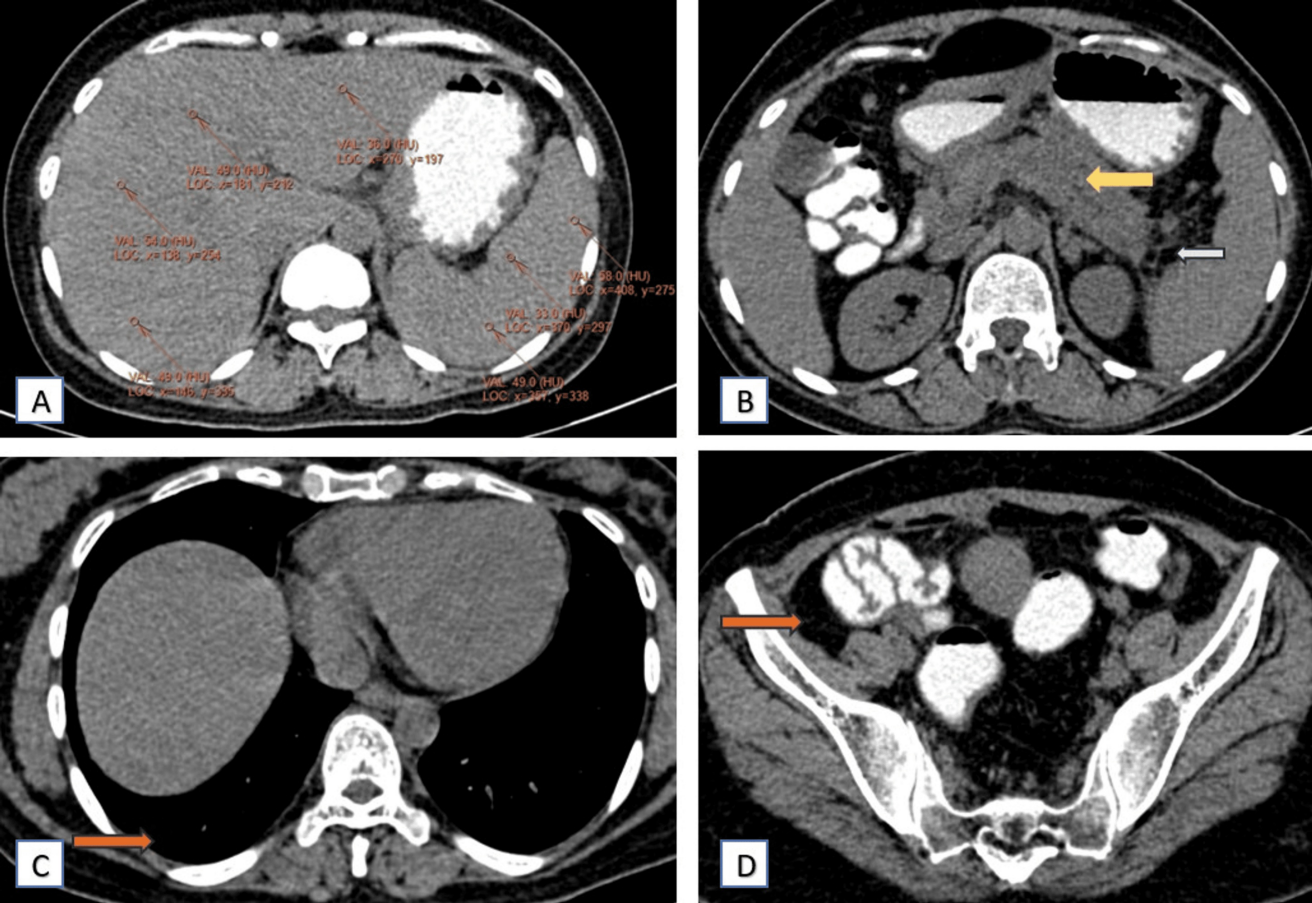
Axial plain with oral contrast CT scan images depicting regression of fatty liver and significant regression of necrotizing pancreatitis, taken after two months of conservative treatment for acute necrotizing pancreatitis Axial plain with oral contrast CT scan images depict normal attenuation of the liver compared to the spleen (A), the pancreas appears normal (yellow arrow) with complete regression of peripancreatic fat stranding and normal left lateral conal fascia (white arrow) (B), complete regression of bilateral pleural effusion (orange arrow) (C), and no free fluid in the pelvis region (orange arrow) (D). CT: computed tomography

**Figure 4:**
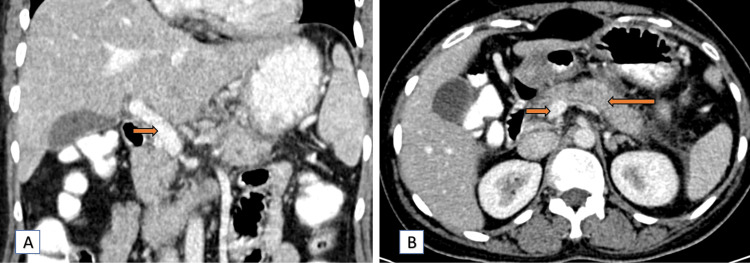
Contrast CT images (coronal and axial) revealing normal contrast opacification in the portal vein and splenic vein Contrast CT images (portal phase) reveal good contrast opacification of the portal vein at the region of the portal vein confluence in coronal (short arrow) (A) and axial (short arrow) (B) sections, with normal pancreatic parenchymal attenuation in the axial (long arrow) (B) section. Contrast CT images (arterial phase) reveal good contrast opacification of the splenic vein in coronal (blue arrow) (C) and axial (blue arrow) (D) sections. CT: computed tomography

Two months later, a follow-up CT scan demonstrated significant improvement. On plain and contrast CT scan, the fatty infiltration in the liver had regressed (Figure 3A), and the necrotizing pancreatitis showed marked improvement (Figure 3B). The peripancreatic inflammation (Figure 3B, 4B), bilateral pleural effusion (Figure 3C), and ascites (Figure 3D) had also reduced significantly, indicating a positive response to the conservative treatment regimen. Post-contrast CT scan, the portal and splenic vein thrombosis showed signs of resolution as well (Figure 4A-4D).

Concurrently, follow-up laboratory studies after two months corroborated the imaging findings (Table 1). There was a notable regression in serum amylase, lipase, C-reactive protein, triglyceride, and D-dimer levels, reflecting a reduction in pancreatic inflammation and systemic inflammatory response. The patient's clinical symptoms had significantly improved, with a marked decrease in abdominal pain and distension, indicating effective management of the acute phase of her illness.

**Table 1:** Pre- and post-treatment comparison of biochemical parameters CRP: C-reactive protein, hs: high sensitivity

Laboratory parameters	Normal levels	On admission	Follow-up after two months of treatment
CRP (hs)	<10 mg/L	287 mg/L	3.22 mg/L
Serum amylase	25-115 U/L	761 U/L	87 U/L
Serum lipase	73-393 U/L	5,455 U/L	135 U/L
Total triglycerides	<150 mg/dL	180 mg/dL	145 mg/dL
D-dimer	0-500 ng/mL	7,272 ng/mL	438 ng/mL

## Discussion

Acute pancreatitis is a severe inflammatory condition that can have systemic repercussions, including developing hepatic fatty liver. The presented case of acute necrotizing pancreatitis (ANP) in a 34-year-old female along with fatty liver underscores the critical nature of this condition and the importance of prompt and comprehensive management. The observed regression of fatty liver infiltration concurrent with improvement in pancreatitis severity, in this case, echoes findings from studies highlighting the interplay between metabolic conditions and pancreatitis outcomes.

Not only the exact mechanism by which acute pancreatitis induces fatty liver is unknown, but there is a lack of literature supporting the impacts of various treatment strategies on reversing hepatic steatosis in ANP patients. Acute pancreatitis and fatty liver can however influence each other significantly. Previous studies have shown a notable correlation between the difference in liver signal intensity on in-phase and out-phase magnetic resonance (MR) images and the MR severity index (MRSI) score in AP patients with fatty liver [12]. The difference in liver signal intensity tends to increase as the MRSI score, a measure used to determine the severity of acute pancreatitis, rises. Further, fatty liver conditions have been reported to improve or even resolve entirely once the patient recovers from AP. This highlights a bidirectional relationship: fatty liver can either develop or worsen during AP, while improvements in AP can lead to the resolution or mitigation of fatty liver [13,14].

A study by Chen et al. (2023) [15] emphasizes that addressing underlying metabolic risk factors, such as obesity and insulin resistance, through lifestyle modifications and potential pharmacotherapy can mitigate the severity of pancreatitis and improve overall liver health outcomes.

Antioxidant therapy has also shown promise in reducing oxidative stress and protecting liver cells in patients with acute pancreatitis. An extensive review by Swentek et al. (2021) [16] discusses the role of antioxidants in managing pancreatitis and consequently improving liver damage. The administration of antioxidants such as vitamin E and N-acetylcysteine can reduce liver damage and promote the resolution of fatty liver. Clinical trials have reported significant improvements in liver enzymes and imaging markers of hepatic steatosis in patients treated with antioxidants [17]. Managing blood glucose levels is another crucial aspect of treating acute pancreatitis and its hepatic complications. Hyperglycemia can worsen fatty liver by increasing lipogenesis and impairing lipid oxidation in the liver [18]. It has been shown that patients with well-controlled blood glucose levels experience faster resolution of liver fat and better overall recovery from pancreatitis [19].

These findings underscore the importance of comprehensive care in managing acute pancreatitis, addressing both the pancreatic inflammation and its systemic effects to achieve optimal patient outcomes.

## Conclusions

The successful management of acute necrotizing pancreatitis (ANP) in this patient demonstrates the critical importance of a comprehensive, evidence-based approach in treating this severe condition. This case also emphasizes the importance of addressing fatty liver disease in conjunction with treating ANP. The observed regression of fatty liver infiltration concurrent with the improvement in pancreatitis severity highlights the interplay between these conditions and the benefits of a holistic treatment approach. This underscores the need for ongoing monitoring and timely intervention to manage both conditions effectively, further supporting the importance of a multidisciplinary approach to optimize patient outcomes.
